# Supplementary material for: Genetic variations of CYP2R1 (rs10741657) in Bangladeshi adults with low serum 25(OH)D level—A pilot study
Source: PLoS One. 2021 Nov 19;16(11):e0260298. doi: 10.1371/journal.pone.0260298 (PMC8604301; doi:10.1371/journal.pone.0260298)

**S_1_ Appendix: Ethical committee and corresponding permissions**

**Ethical clearance certificate**


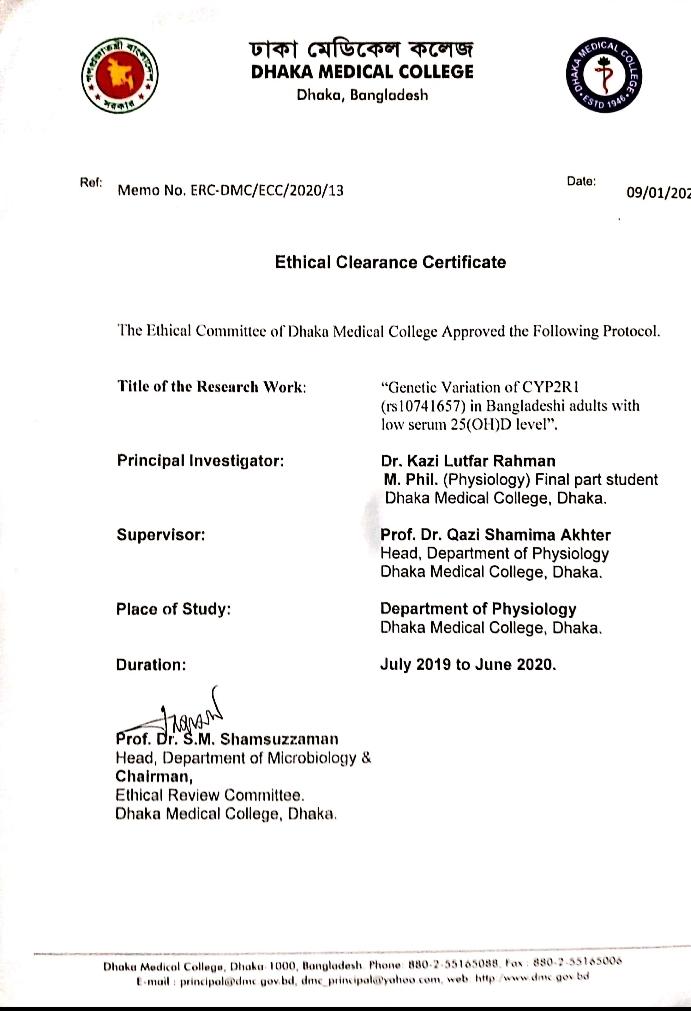


**Permission letter from Center for medical Biotechnology**


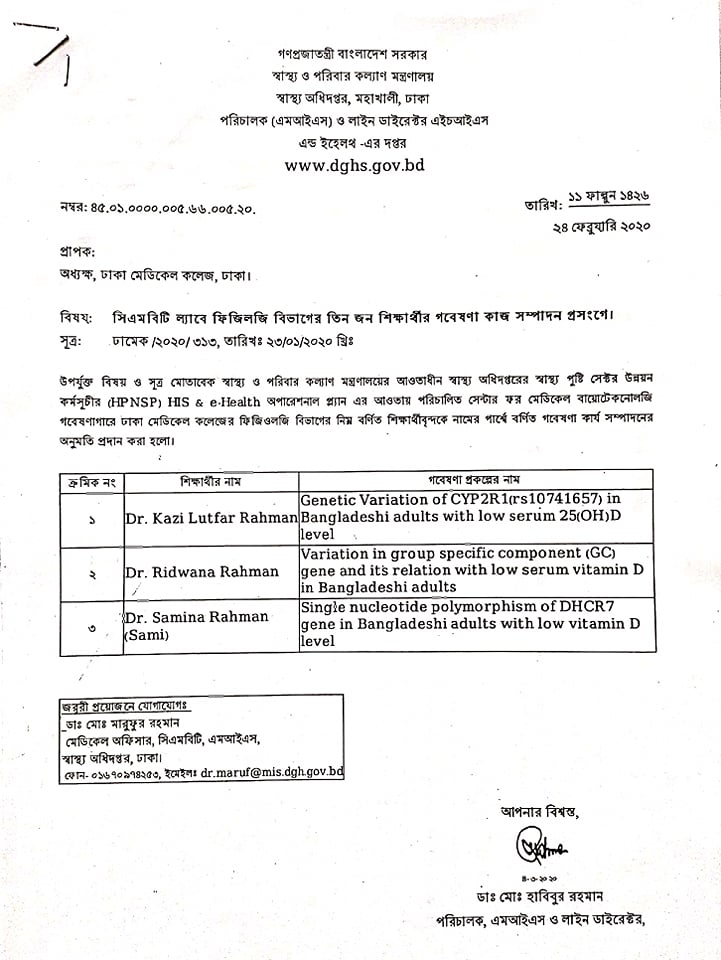


**Permission letter from Dhaka Metropolitan Police**


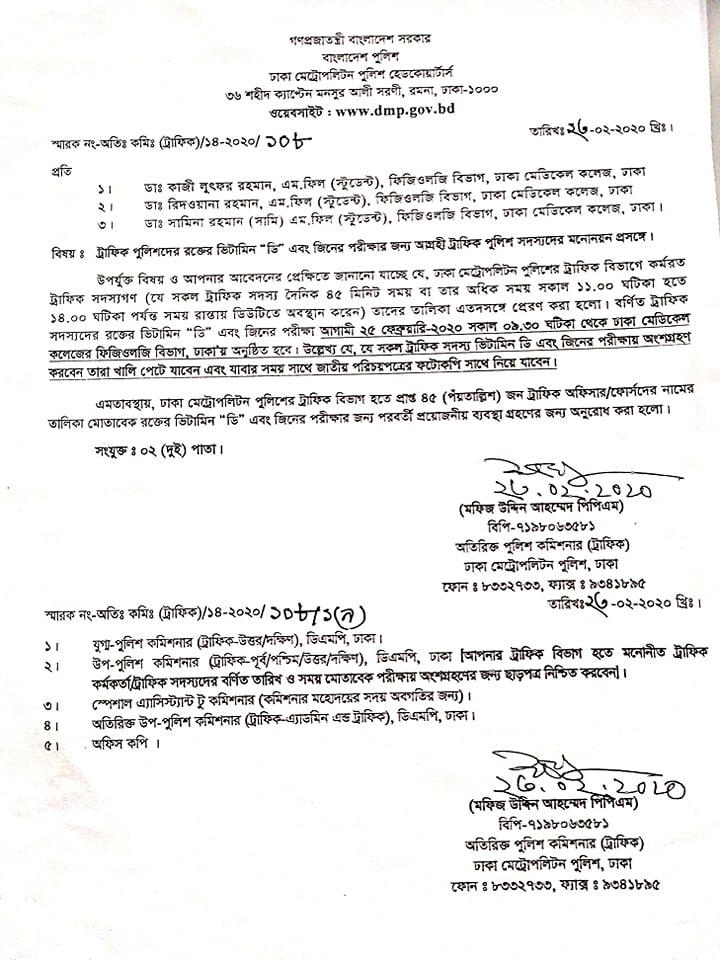


**Permission letter from Good Neighbors Bangladesh NGO**


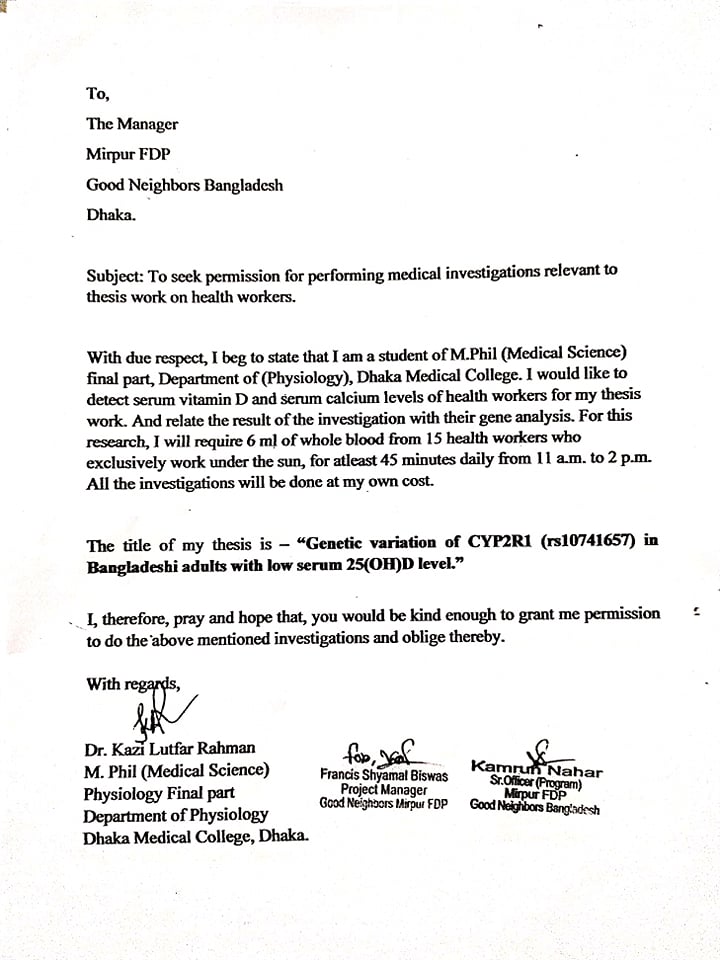


# Permission letter from Clinical Pathology, Dhaka Medical College


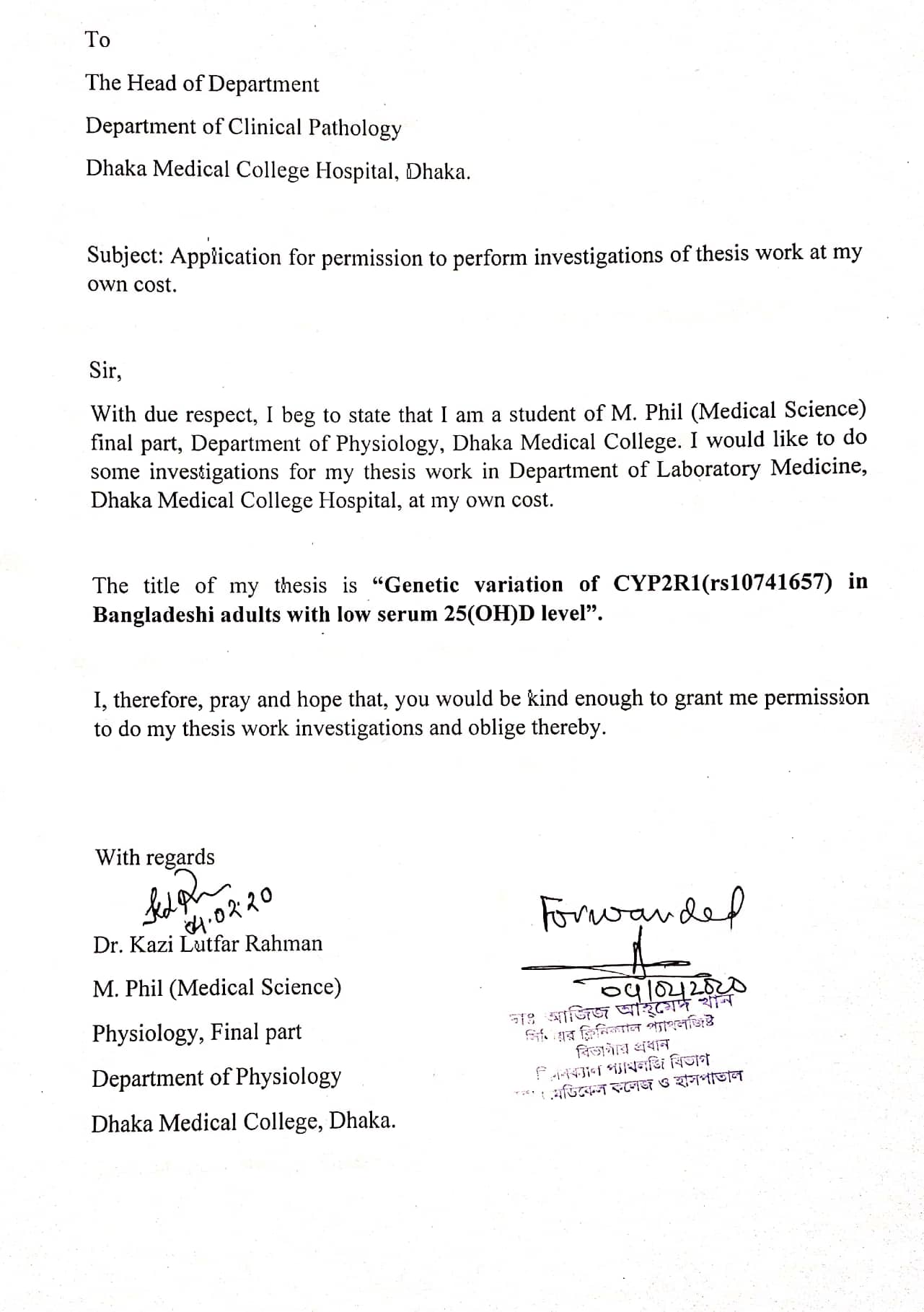


**Permission letter from Director, Dhaka Medical College**


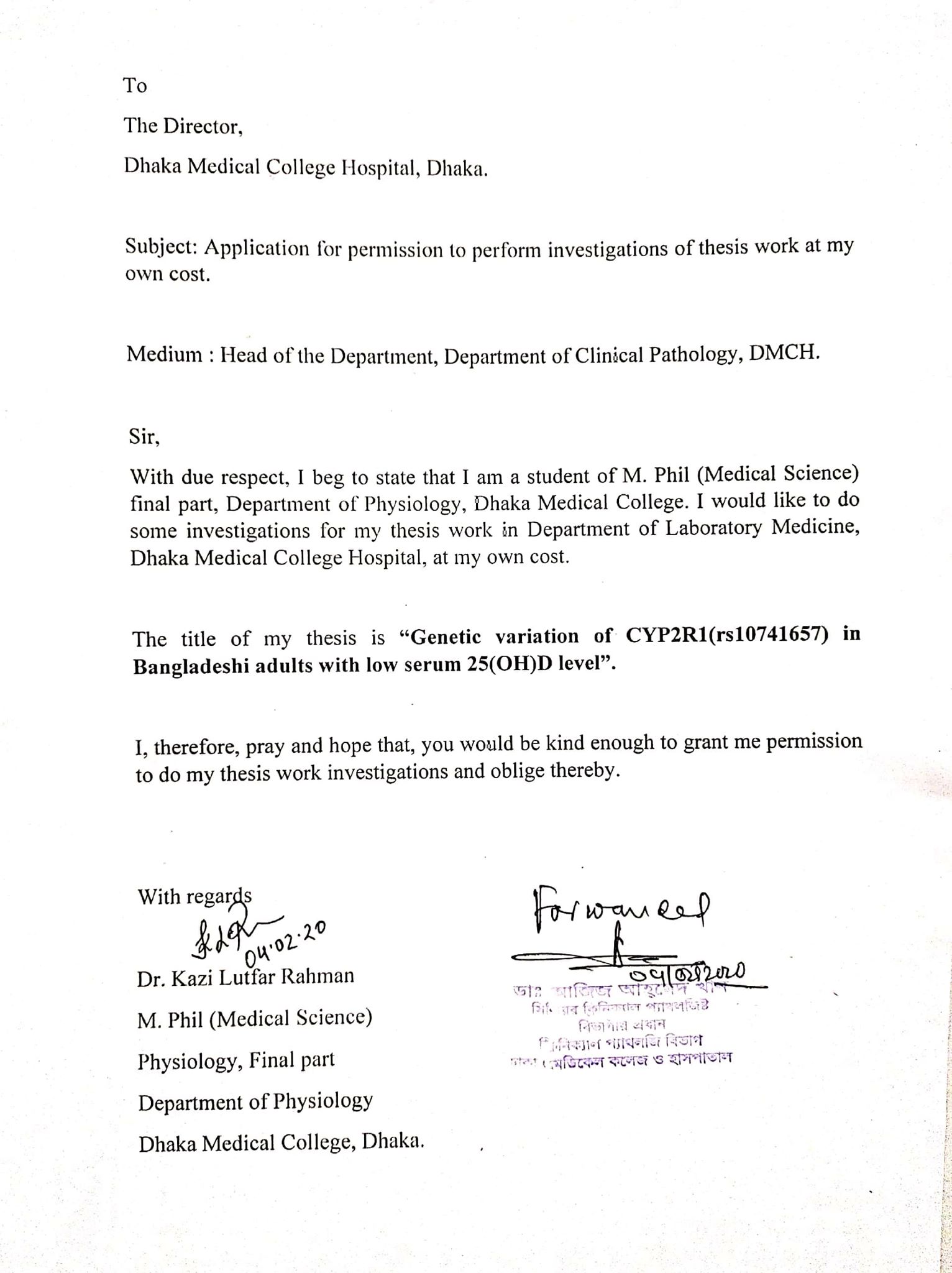

Supplement: S1 Appendix — (DOCX) [file pone.0260298.s001.docx]
