# Supplementary material for: Genetic variations of CYP2R1 (rs10741657) in Bangladeshi adults with low serum 25(OH)D level—A pilot study
Source: PLoS One. 2021 Nov 19;16(11):e0260298. doi: 10.1371/journal.pone.0260298 (PMC8604301; doi:10.1371/journal.pone.0260298)
Supplement: S2 Appendix — (DOCX) [file pone.0260298.s002.docx]

**S_2_ Appendix: NCBI extension numbers of Sequence files**

All the sequencing files of study subjects were submitted on NCBI, which were accepted with valid extension numbers on NCBI, ENA and GenBank of JAPAN, here all the extension numbers with NCBI links are provided for further assessment if required.

**Sequencing files extension numbers and links of study population:**

Nucleotide extension number: MT724707

Link: https://www.ncbi.nlm.nih.gov/nuccore/MT724707

Nucleotide extension number: MT724708

Link: https://www.ncbi.nlm.nih.gov/nuccore/MT724708

Nucleotide extension number: MT724709

Link: https://www.ncbi.nlm.nih.gov/nuccore/MT724709

Nucleotide extension number: MT724710

Link: https://www.ncbi.nlm.nih.gov/nuccore/MT724710

Nucleotide extension number: MT724711

Link: https://www.ncbi.nlm.nih.gov/nuccore/MT724711

Nucleotide extension number: MT724712

Link: https://www.ncbi.nlm.nih.gov/nuccore/MT724712

Nucleotide extension number: MT724713

Link: https://www.ncbi.nlm.nih.gov/nuccore/MT724713

Nucleotide extension number: MT724714

Link: https://www.ncbi.nlm.nih.gov/nuccore/MT724714

Nucleotide extension number: MT724715

Link: https://www.ncbi.nlm.nih.gov/nuccore/MT724715

Nucleotide extension number: MT724716

Link: https://www.ncbi.nlm.nih.gov/nuccore/MT724716

Nucleotide extension number: MT724717

Link: https://www.ncbi.nlm.nih.gov/nuccore/MT724717

Nucleotide extension number: MT724718

Link: https://www.ncbi.nlm.nih.gov/nuccore/MT724718

Nucleotide extension number: MT724719

Link: https://www.ncbi.nlm.nih.gov/nuccore/MT724719

Nucleotide extension number: MT724720

Link: https://www.ncbi.nlm.nih.gov/nuccore/MT724720

Nucleotide extension number: MT724721

Link: https://www.ncbi.nlm.nih.gov/nuccore/MT724721

Nucleotide extension number: MT724722

Link: https://www.ncbi.nlm.nih.gov/nuccore/MT724722

Nucleotide extension number: MT724723

Link: https://www.ncbi.nlm.nih.gov/nuccore/MT724723

Nucleotide extension number: MT724724

Link: https://www.ncbi.nlm.nih.gov/nuccore/MT724724

Nucleotide extension number: MT724725

Link: https://www.ncbi.nlm.nih.gov/nuccore/MT724725

Nucleotide extension number: MT724726

Link: https://www.ncbi.nlm.nih.gov/nuccore/MT724726

Nucleotide extension number: MT724727

Link: https://www.ncbi.nlm.nih.gov/nuccore/MT724727

Nucleotide extension number: MT724728

Link: https://www.ncbi.nlm.nih.gov/nuccore/MT724728

Nucleotide extension number: MT724729

Link: https://www.ncbi.nlm.nih.gov/nuccore/MT724729

Nucleotide extension number: MT724730

Link: https://www.ncbi.nlm.nih.gov/nuccore/MT724730

Nucleotide extension number: MT724731

Link: https://www.ncbi.nlm.nih.gov/nuccore/MT724731

Nucleotide extension number: MT724732

Link: https://www.ncbi.nlm.nih.gov/nuccore/MT724732

Nucleotide extension number: MT724733

Link: https://www.ncbi.nlm.nih.gov/nuccore/MT724733

Nucleotide extension number: MT724734

Link: https://www.ncbi.nlm.nih.gov/nuccore/MT724734

Nucleotide extension number: MT724735

Link: https://www.ncbi.nlm.nih.gov/nuccore/MT724735

Nucleotide extension number: MT724736

Link: https://www.ncbi.nlm.nih.gov/nuccore/MT724736

**Sequencing files extension numbers and links of controls:**

Nucleotide extension number: MT724737

Link: https://www.ncbi.nlm.nih.gov/nuccore/MT724737

Nucleotide extension number: MT724740

Link: https://www.ncbi.nlm.nih.gov/nuccore/MT724740

Nucleotide extension number: MT724741

Link: https://www.ncbi.nlm.nih.gov/nuccore/MT724741

Nucleotide extension number: MT724742

Link: https://www.ncbi.nlm.nih.gov/nuccore/MT724742

Nucleotide extension number: MT724743

Link: https://www.ncbi.nlm.nih.gov/nuccore/MT724743

Nucleotide extension number: MT724744

Link: https://www.ncbi.nlm.nih.gov/nuccore/MT724744

Nucleotide extension number: MT724745

Link: https://www.ncbi.nlm.nih.gov/nuccore/MT724745

Nucleotide extension number: MT724746

Link: https://www.ncbi.nlm.nih.gov/nuccore/MT724746

Nucleotide extension number: MT724747

Link: https://www.ncbi.nlm.nih.gov/nuccore/MT724747

Nucleotide extension number: MT724748

Link: https://www.ncbi.nlm.nih.gov/nuccore/MT724748
