# Supplementary material for: Genetic variations of CYP2R1 (rs10741657) in Bangladeshi adults with low serum 25(OH)D level—A pilot study
Source: PLoS One. 2021 Nov 19;16(11):e0260298. doi: 10.1371/journal.pone.0260298 (PMC8604301; doi:10.1371/journal.pone.0260298)
Supplement: S1 File — (DOCX) [file pone.0260298.s003.docx]

| **Available Data of the study subjects**  **Sociodemographic and laboratory raw Data of study population** | | | | | | | | | | | | | | | | |
| --- | --- | --- | --- | --- | --- | --- | --- | --- | --- | --- | --- | --- | --- | --- | --- | --- |
| Sl. | Co-de | Age  (ye-ars) | Sex | Profession | SBP  (mm of Hg) | DBP  (mm of Hg) | Duration  Of sun Exposure | Serum 25(OH)D level (ng/ml) | Genotypes of rs10741657 | Serum Calcium level  (mg/dl) | Serum Creatin-ine level  (mg/dl) | SGPT  (U/L) | Serum Albumin  level  (gm/dl) | Fasting Blood Sugar  (mmol/L) | Proth-rombin Time  (Sec) | BMI  (Kg/m^2^) |
| 1 | P3 | 42 | M | Police | 120 | 70 | 8 hours | 22.48 | GG | 8.67 | 1.13 | 16 | 4.37 | 5.01 | 12.2 | 23.70 |
| 2 | P5 | 51 | M | Police | 125 | 75 | 8 hours | 23.13 | GG | 9.22 | 1.17 | 22 | 4.91 | 4.32 | 12.4 | 23.50 |
| 3 | P7 | 36 | M | Police | 110 | 75 | 8 hours | 24.18 | GG | 8.53 | 0.96 | 17 | 4.34 | 4.78 | 12.4 | 24.50 |
| 4 | P10 | 44 | M | Police | 100 | 85 | 8 hours | 23.50 | GG | 8.78 | 1.21 | 33 | 4.91 | 3.99 | 12.0 | 22.80 |
| 5 | P16 | 53 | M | Police | 115 | 70 | 8 hours | 21.21 | GG | 9.11 | 1.14 | 16 | 4.33 | 4.21 | 12.7 | 24.20 |
| 6 | P18 | 53 | M | Police | 120 | 65 | 8 hours | 24.78 | GG | 8.63 | 1.24 | 15 | 4.38 | 5.12 | 12.2 | 20.60 |
| 7 | P19 | 50 | M | Police | 115 | 80 | 8 hours | 26.37 | GG | 9.21 | 1.18 | 17 | 4.29 | 5.37 | 12.1 | 22.10 |
| 8 | P27 | 44 | M | Police | 110 | 80 | 8 hours | 26.24 | GG | 8.54 | 1.29 | 12 | 4.10 | 5.41 | 12.0 | 22.80 |
| 9 | P26 | 48 | M | Police | 115 | 65 | 8 hours | 23.48 | GG | 8.99 | 1.34 | 18 | 4.99 | 4.58 | 12.5 | 23.80 |
| 10 | P29 | 46 | M | Police | 100 | 60 | 8 hours | 22.79 | GG | 8.90 | 1.11 | 29 | 4.29 | 4.12 | 12.5 | 23.80 |
| 11 | P30 | 42 | M | Police | 120 | 85 | 4 hours | 26.20 | GA | 8.67 | 0.93 | 21 | 3.78 | 5.95 | 13.2 | 22.50 |
| 12 | P33 | 45 | M | Police | 115 | 75 | 8 hours | 28.12 | GA | 9.85 | 1.14 | 19 | 4.21 | 3.91 | 12.8 | 21.20 |
| 13 | P35 | 39 | M | police | 120 | 75 | 8 hours | 22.55 | GA | 8.91 | 0.89 | 12 | 3.89 | 4.51 | 12.5 | 22.10 |
| 14 | P36 | 39 | M | Police | 115 | 80 | 7 hours | 24.88 | GA | 9.11 | 0.99 | 17 | 4.17 | 4.29 | 13.5 | 23.80 |
| 15 | G14 | 40 | F | H. W. | 100 | 75 | 7 hours | 23.15 | GG | 9.23 | 0.76 | 19 | 4.98 | 3.98 | 12.0 | 23.00 |
| 16 | G13 | 43 | F | H.W. | 120 | 70 | 4 hours | 25.36 | AA | 9.75 | 0.89 | 16 | 3.57 | 4.27 | 15.0 | 21.90 |
| 17 | G9 | 45 | F | H.W. | 115 | 85 | 3 hours | 22.48 | GG | 8.85 | 0.93 | 10 | 4.18 | 4.11 | 12.0 | 21.70 |
| 18 | G5 | 35 | F | H.W. | 120 | 80 | 4 hours | 25.29 | GG | 8.88 | 0.67 | 27 | 3.99 | 4.63 | 12.0 | 22.80 |
| 19 | G2 | 33 | F | H.W. | 125 | 70 | 6 hours | 27.92 | GG | 8.52 | 0.87 | 28 | 4.14 | 4.54 | 12.0 | 20.30 |
| 20 | A15 | 18 | M | Player | 110 | 80 | 6 hours | 26.21 | GA | 9.12 | 1.15 | 13 | 4.16 | 4.34 | 12.3 | 22.80 |
| 21 | A24 | 18 | M | Player | 115 | 80 | 7 hours | 28.99 | GA | 9.24 | 1.12 | 16 | 3.87 | 3.94 | 12.4 | 22.70 |
| 22 | A20 | 19 | M | Player | 120 | 75 | 4 hours | 24.56 | GA | 9.55 | 0.89 | 13 | 3.29 | 4.74 | 12.0 | 23.50 |
| 23 | A22 | 20 | M | Player | 130 | 80 | 4 hours | 28.34 | GA | 9.76 | 0.87 | 19 | 4.89 | 4.38 | 12.3 | 21.20 |
| 24 | A26 | 20 | M | Player | 125 | 70 | 4 hours | 28.76 | GA | 9.23 | 0.82 | 17 | 4.94 | 4.32 | 12.8 | 24.10 |
| 25 | A4 | 22 | M | Player | 120 | 70 | 4 hours | 27.91 | AA | 9.87 | 0.97 | 13 | 4.39 | 3.93 | 13.2 | 22.10 |
| 26 | A5 | 21 | M | Player | 120 | 75 | 5 hours | 24.50 | GG | 9.12 | 1.24 | 13 | 4.11 | 4.47 | 13.0 | 23.50 |
| 27 | A8 | 18 | M | Player | 115 | 80 | 7 hours | 24.28 | GG | 9.56 | 0.97 | 18 | 4.18 | 3.92 | 12.0 | 22.10 |
| 28 | A9 | 18 | M | Player | 120 | 75 | 6 hours | 27.52 | GG | 9.34 | 1.18 | 12 | 4.95 | 4.57 | 14.2 | 22.10 |
| 29 | A10 | 18 | M | Player | 120 | 75 | 7 hours | 23.96 | GG | 8.75 | 1.54 | 11 | 3.99 | 5.14 | 13.8 | 22.10 |
| 30 | A13 | 18 | M | Player | 110 | 85 | 6 hours | 26.89 | GG | 9.14 | 0.91 | 17 | 4.87 | 3.94 | 12.2 | 20.50 |
| **Sociodemographic and laboratory raw Data of Controls** | | | | | | | | | | | | | | | | |
| Sl. | Co-de | Age | Sex | Profession | SBP  (mm of Hg) | DBP  (mm of Hg) | Duration  Of sun Exposure | Serum 25(OH)D level (ng/ml) | Genotypes of rs10741657 | Serum Calcium level  (mg/dl) | Serum Creatinine level  (mg/dl) | SGPT  (U/L) | Serum Albumin  level  (gm/dl) | Fasting Blood Sugar  (mmol/L) | Prothrombin Time  (Sec) | BMI  (Kg/m^2^) |
| 1 | P38 | 44 | M | Police | 115 | 70.00 | 8 hours | 34.23 | GA | 9.11 | 1.25 | 14 | 3.80 | 5.11 | 14.8 | 23.8 |
| 2 | G18 | 30 | F | H. W. | 110 | 70.00 | 2 hours | 41.38 | GA | 9.89 | 0.97 | 17 | 4.41 | 4.26 | 15.2 | 22.3 |
| 3 | A27 | 18 | M | Player | 120 | 85.00 | 5 hours | 35.74 | GG | 9.23 | 1.13 | 21 | 4.37 | 5.25 | 12.5 | 22.1 |
| 4 | A29 | 19 | M | Player | 120 | 75.00 | 6 hours | 38.57 | GA | 9.07 | 1.07 | 34 | 3.99 | 4.67 | 12.0 | 23.5 |
| 5 | A35 | 19 | M | Player | 120 | 70.00 | 6 hours | 72.95 | GA | 9.98 | 0.98 | 21 | 4.11 | 4.32 | 14.5 | 22.4 |
| 6 | R1 | 25 | M | Player | 110 | 80.00 | 2 hours | 39.25 | GG | 9.47 | 1.23 | 17 | 3.77 | 5.22 | 12.0 | 23.1 |
| 7 | R3 | 25 | M | Player | 115 | 80.00 | 2 hours | 41.39 | AA | 9.21 | 0.88 | 27 | 4.39 | 4.67 | 12.0 | 21.4 |
| 8 | P39 | 40 | M | Police | 115 | 85.00 | 8 hours | 67.52 | AA | 10.12 | 0.71 | 26 | 4.11 | 4.88 | 13.0 | 20.5 |
| 9 | G19 | 37 | F | H.W. | 110 | 75.00 | 2 hours | 55.91 | GA | 9.21 | 0.94 | 21 | 3.50 | 5.21 | 14.0 | 21.5 |
| 10 | G26 | 32 | F | H.W. | 110 | 80.00 | 2 hours | 69.49 | AA | 9.88 | 0.82 | 28 | 4.18 | 4.87 | 15.0 | 21.7 |

**Sociodemographic and laboratory raw Data of the people who were excluded from the current study**

| Sl. | Code | Age  (Years) | Sex | Profession | SBP  (mm of Hg) | DBP  (mm of Hg) | Duration  Of sun Exposure | Serum 25(OH)D level (ng/ml) | Genotypes of rs10741657 | Serum Calcium level  (mg/dl) | Serum Creatinine level  (mg/dl) | SGPT  (U/L) | Serum Albumin  level  (gm/dl) | Fasting Blood Sugar  (mmol/L) | Prothrombin Time  (Sec) | BMI  (Kg/m^2^) |
| --- | --- | --- | --- | --- | --- | --- | --- | --- | --- | --- | --- | --- | --- | --- | --- | --- |
| 1 | G1 | 31 | F | H. W | 120 | 70 | 2 hours | 33.96 | Not done | 9.21 | 0.74 | 16 | 3.39 | 4.69 | 12.5 | 21.1 |
| 2 | G3 | 28 | F | H. W | 120 | 60 | 2 hours | 32.18 | Not done | 9.57 | 0.66 | 39 | 3.21 | 3.98 | 13.5 | 20.8 |
| 3 | G4 | 23 | F | H. W | 125 | 65 | 2 hours | 37.19 | Not done | 8.69 | 0.89 | 15 | 4.26 | 5.17 | 16.0 | 22.2 |
| 4 | G6 | 57 | F | H.W. | 100 | 65 | 2 hours | 34.53 | Not done | 8.89 | 0.69 | 39 | 4.67 | 4.58 | 15.5 | 23.7 |
| 5 | A1 | 24 | M | Player | 120 | 80 | 6 hours | 41.24 | Not done | 9.14 | 0.94 | 21 | 3.89 | 4.21 | 15.5 | 22.8 |
| 6 | A2 | 28 | M | Player | 120 | 85 | 6 hours | 30.29 | Not done | 9.17 | 1.17 | 17 | 3.45 | 4.19 | 12.0 | 21.8 |
| 7 | A3 | 28 | M | Player | 130 | 85 | 6 hours | 33.91 | Not done | 9.58 | 1.15 | 19 | 4.16 | 4.28 | 12.4 | 21.4 |
| 8 | A6 | 20 | M | Player | 120 | 70 | 7 hours | 41.37 | Not done | 9.10 | 1.77 | 25 | 3.50 | 5.14 | 13.0 | 20.6 |
| 9 | G7 | 33 | F | H.W. | 120 | 7 | 2 hours | 30.99 | Not done | 9.58 | 1.07 | 31 | 3.87 | 5.19 | 14.0 | 21.4 |
| 10 | G8 | 24 | F | H.W. | 130 | 0 | 2 hours | 33.36 | Not done | 9.47 | 0.68 | 17 | 3.37 | 5.99 | 13.0 | 21.1 |
| 11 | A7 | 24 | M | Player | 125 | 75 | 6 hours | 37.51 | Not done | 8.90 | 0.97 | 19 | 3.68 | 5.13 | 12.0 | 20.5 |
| 12 | A11 | 19 | M | Player | 115 | 85 | 6 hours | 33.79 | Not done | 9.12 | 1.16 | 21 | 3.99 | 4.99 | 14.0 | 22.8 |
| 13 | A12 | 24 | M | Player | 125 | 85 | 6 hours | 32.53 | Not done | 8.57 | 0.98 | 28 | 4.25 | 3.96 | 15.0 | 22.1 |
| 14 | A14 | 19 | M | Player | 1115 | 80 | 5 hours | 34.07 | Not done | 9.11 | 1.65 | 17 | 3.11 | 4.29 | 12.0 | 21.8 |

Note: H. W= Health workers (community NGO worker) ; M= male; F= Female, SBP= Systolic Blood Pressure; DBP= Diastolic Blood pressure
