# Supplementary material for: Genetic variations of CYP2R1 (rs10741657) in Bangladeshi adults with low serum 25(OH)D level—A pilot study
Source: PLoS One. 2021 Nov 19;16(11):e0260298. doi: 10.1371/journal.pone.0260298 (PMC8604301; doi:10.1371/journal.pone.0260298)
Supplement: Attachment — Submitted filename: Response to Reviewers.docx [file pone.0260298.s004.docx]

September 29, 2021

Editor-in-Chief

PLOS ONE

Dear Editor-in-Chief,

I am submitting the 1^st^ revised version of the manuscript titled “**Genetic Variation of CYP2R1 (rs10741657) in Bangladeshi adults with low serum 25(OH)D level**” to PLOS ONE.

I really appreciate all of your efforts and constructive criticisms which will level up our current manuscript. I have carefully gone through each of the comments from the peer reviewers on our original manuscript. As the current study was a pilot study in Bangladesh with a small sample size, we have modified the title to “**Genetic Variation of *CYP2R1* (rs10741657) in Bangladeshi adults with low serum 25(OH)D level-A Pilot Study**”.

Here I am trying my best to answer all the questions and concerns which were raised by the academic editor and reviewers.

Responses to academic editor

- Please, follow appropriate reporting guidelines, such as STROBE

Response: Thank you for your kind concern. In this 1^st^ revised version of the manuscript, we have tried to maintain it accordingly.

- Please, better specify the objectives of the study; it would be better to add the word to determine “frequency” of variants…)

Response: This has been done in the current version of the manuscript. Please check page no. 2, line no. 33 and page no. 2, line no. 84 to 88.

- The small sample size is a major limitation. Authors stated that this is a pilot study (this should be better also included in the title, discussion, and conclusion).

Response: Thank you so much, it was really needed. This has been done in the current version of the manuscript. Please check page no. 1, line no. 5, and page no. 13, line no. 258 and page no. 16, line no. 326.

Regarding Data availability, authors stated “Yes - all data are fully available without restriction” and data are “within the manuscript and its as supporting information files”. However, the submission doesn’t contain unidentified patients’ data. According to PLOS ONE publication criteria, authors are required to make all data underlying the findings described fully available, without restriction, and from the time of publication. PLOS allows rare exceptions to address legal and ethical concerns, that should be clearly explained.

Response: All the data are now uploaded in a new file, but the details of the study subjects are hidden to maintain their privacy. All the gene data with NCBI links are already given in the previous submission.

- Authors should clearly describe participants’ inclusion and exclusion criteria, time of recruitment, how participants were selected (convenience sample?)

Response: This has been done in the current version of the manuscript. Please check page no. 4, line no. 92 to page no. 5, line no. 112.

- Authors should provide definitions/diagnostic criteria for described conditions/outcomes, including vitamin D deficiency.

Response: This has been done in the current version of the manuscript. Please check page no. 3, line no. 58 to 60 and page no. 6, line 118 to 122.

- The authors didn’t study other genetic determinants (other vitamin D associated genes), which could be possible confounders.

Response: This was the limitation of the study. As it was a part of an academic research project, we could not analyze other genetic determinants due to time and financial constraints. In addition, this has been explained in the current version of the manuscript. Please check page no.16, line no. 318 to 323 and page no.17, line no. 338 to 341.

- In statistical analysis, were data tested for normality (some data are reported as mean (SD) but appear not normally distributed). What about confounders?

Response: Yes, all data were tested for normality by the normal Q- Q plot of Shapiro- Wilk Normality Test (SPSS version 23 software). Mean (SD) of age, BMI, FBS, serum Ca^++^, creatinine, SGPT and albumin level of the study subjects and serum 25(OH)D level of the study population were apparently normally distributed. But serum 25(OH)D level of the controls were not normally distributed. The confounders could not be clearly understood for the mean serum 25(OH)D level of the controls as the number of the controls was only 10 and other genetic determinants could not be analyzed due to financial constraints.

- Study results and tables should be reported in the results section.

Response: This has been done in the current version of the manuscript. Please check page no. 9, line no. 195 and page no. 10, line no. 206.

- Tables require better organization with comparison of variables between the study and control groups.

Response: This has been done in the current version of the manuscript. Please check page no. 9, line no. 195, page no. 10, line no. 206 and page no. 13, line no. 245.

- In figure 1, it would be better to annotate the polymorphisms.

Response: This has been done in the current version of the manuscript.

- Figures 2 & 3 seem to be of little value as its data can be incorporated in tables or text.

Response: Figures 2 & 3 have been replaced by new figures and the previous data are incorporated in text in the current version.

- Discussion requires major revision, critically discussing main findings of study (without repeating results) and study implications.

Response: This has been done in the current version of the manuscript.

- In light of being a pilot, the current conclusion seems to overestimate study findings; authors should reduce the strength of their statement, confirming that this is a pilot study, and recommend further well-powered studies.

Response: Those have been changed in the current version. Please check page no. 16, line no. 326 to page no. 17, line no. 334.

- Please, revise the references to follow PLOS ONE style.

Response: This has been done in the current version.

- Authors should revise the manuscript for several errors in language, sentence structure, grammar, and punctuation.

Response: We have tried our best to follow proper language, sentence structure, grammar and punctuation in the current manuscript.

Responses to Reviewer#1

- I have a major concern regarding the sample size of the study. With the current small sample size and the used study design, the study probably is underpowered. Was the sample size and power of the study was calculated prior starting the study?

Response: Thank you for your kind concern. This was a pilot study and none of the vitamin D influencing gene has ever been analyzed in Bangladesh. Moreover, as it was an academic research project, only 30 subjects as study population with low serum 25(OH)D level and 10 subjects sufficient serum 25(OH)D level as controls were included due to time and financial constraints. Therefore, we could not calculate the sample size and power of the study prior to the research.

- In line 73 (in the introduction), Please remove DBP as GC is already mentioned and It is the gene encoding vitamin D binding protein (DBP). In addition, please include CYP27B1 gene with the other major common vitamin D genes as it is one of the significant genes involved in vitamin D metabolism (it is the gene encoding 1α-hydroxylase, the enzyme responsible for the 2nd step of activation of D in the kidney).

Response: This has been done in the current version of the manuscript. Please check page no. 4, line no. 74.

- Please mention the exclusion and inclusion criteria in the methods and the inter and intra-assay CV for the performed tests.

Response: This has been done in the current version of the manuscript. Please check page no. 5 line no. 98 to 112 and page no. 6, line no. 131 and 132. Except serum 25(OH)D level, the intra and inter assay CV of other parameters were not estimated. Other samples were analyzed for a single time as there was a scarcity of laboratory reagents due to financial constraints. This is because serum 25(OH)D level and genetic variants of CYP2R1 (rs10741657) were our study parameter and other parameters were estimated to confirm only the exclusion and inclusion criteria.

- Please move Table 1 and 2 to the Results section.

Response: This has been done in the current version of the manuscript. Please check page no. 9, line no. 195 and page no. 10, line no. 206.

- Information in line 186-187 (in the results) is already mentioned in the methods, please remove.

Response: This has been removed in the current version of the manuscript.

Responses to Reviewer#2

- First of all, I would like to congratulate the researchers for this very good work. The "CONCLUSION" section is also finished with a very good conclusion. However, the deficiency that I see in the study and other similar studies is that it is in the form of statistically researching the negativities associated with vitamin D deficiency and making a connection between them. This should provide an advantage if a selective mutation is dominant in geographic regions where this mutation is present. What advantage does vitamin d provide at lower levels? What protection does it provide for people in those areas? The answer to this question should also be explored in the comments. Natural selection never happens without a reason. When I look at it from the point of view of the country I live in, kidney stones come to mind. But I think this should be investigated. If researchers can add an answer and comment to this question in terms of their own country, I think that their work will be more beneficial than other studies. Beautiful original work worth publishing. The journal meets all publication criteria. I wish the researchers success in their new work.

Response: Thank you so much for your kind consideration. Actually, Bangladesh is a developing country and execution of such molecular research projects of any level is quite challenging. You may understand by the fact that this is the first genetic analysis which was carried out in Dhaka Medical College, Bangladesh. As, it was an academic study, it was to complete the whole research work in time considering the recent pandemic. Hence, your kind notes of appreciation have encouraged us to continue in this field of research in future.

The aforementioned concerns have been included in the current version of the revised manuscript. Please check page no. 15 to 17.

Responses to Reviewer#3

- Grammar and sentence construction i nthe entire manuscript have to be revised/ improved

Response: Thank you for your kind concern. This has been revised in the current version.

- What is the significance of yorus tidu if this has been reported in the literature already?

Response: This study has never been conducted in Bangladesh. As a pilot study, our goal was to determine the variants of CYP2R1 (rs10741657) gene and their association with low serum vitamin D level among Bangladeshi adults. The objectives have been elaborated in the current version, please check page no. 4, line no. 80 to 88.

- inclusion and exclusion criteria not stated

Response: This has been stated in the current version of the manuscript. Please check page no. 5, line no. 98 to page no.6, line no 122.

- how di you come up with the final nuber of subjects and how did you choose the initial of 54. please state clearly how you got yoru subjects

Response: Thank you so much again to give us a chance to clear about the fact. This was a pilot study and none of the vitamin D influencing gene has ever been analyzed in Bangladesh. Moreover, as it was an academic research project, only 30 subjects as study population with low serum 25(OH)D level and 10 subjects sufficient serum 25(OH)D level as controls were included due to time and financial constraints. This has been explained in the current version, please check page no. 6, line no. 122 to 136, and page no. 5, line no. 98 to 114.

- what was the significantce of all the other biochemical parameters that were taken apart form vitamin D levels

Response: This has been explained in the current version, please check page no. 5, line no. 107 to 111.

- for tables 1 and 2 make them reader friendly-remove too many lines and make it cleaner. yo ulaso did not specify the statistical significance betwenn the two groups of all the parameters

Response: This has been done in the current version of the manuscript. Please check page no. 9, line no. 195 and page no. 10, line no. 206.

- for table 3 you did not compare the results between groups. you only compared them within the groyp. it wuld be ebtter if you compared for exampe the genoype GG of affected and controls and the corresponding statistical significance

Response: Thank you for your concern, in table 3, only 2 controls had genotype GG, so the comparison between the groups was not done. In addition, the sample size was small and we took only 10 controls to see if there was any variation among the genotypes and alleles between study population and controls. For your kind concern we have added a new table to explain it. This has been explained in page no. 13, line no. 245 to 254 (Table 4).

- i nthe discussion, do not repeat anymore your results but instead interpret and explain them. your tables hsould be self explanatory and in the discussion you will only give its importance

Response: Thank you so much for your kind advice. This has been corrected in the current version.

- what is the impact of the study?

Response: This has been explained in the current version. Please check page no. 16, line no. 313 to page no. 17, line no. 334.

- what is the implciation i nterms of treatment?

Response: This has been explained in the current version. Please check page no. 16, line no. 318 to page no. 17, line no. 334.

- make the discussion mroe concise

Response: This has been corrected in the current version accordingly. Please check page no. line no.

Thank you very much for considering our manuscript for revision.

Yours Sincerely,

Kazi Lutfar Rahman

Email: klrahman@yahoo.com
