# Supplementary material for: Genetic variations of CYP2R1 (rs10741657) in Bangladeshi adults with low serum 25(OH)D level—A pilot study
Source: PLoS One. 2021 Nov 19;16(11):e0260298. doi: 10.1371/journal.pone.0260298 (PMC8604301; doi:10.1371/journal.pone.0260298)
Supplement: Attachment — Submitted filename: Response to Reviewers.docx [file pone.0260298.s005.docx]

October 16, 2021

Editor-in-Chief

PLOS ONE

Dear Editor-in-Chief,

I am submitting the 2^nd^ revised version of the manuscript titled “**Genetic Variation of *CYP2R1* (rs10741657) in Bangladeshi adults with low serum 25(OH)D level-A Pilot Study**” to PLOS ONE.

My wholehearted gratitude goes to the reviewers for whom an improved version of this manuscript could be achieved.

Here I am trying my best to answer all the questions and concerns which were raised by the academic editor and reviewers.

Responses to academic editor

- You are kindly requested to respond to some concerns raised by Reviewer #3. Moreover, authors should briefly emphasize, in the results section, most important findings in the table (not just saying data are shown in tables). Last, the limitations should be moved to appear before the conclusion (could be integrated with the last paragraph in the discussion)

Response: Thank you so much for your appreciation of the revised version. All the concerns raised by Reviewer #3 are responded accordingly in this current version of the manuscript. Most important findings of the table are emphasized in the current version, please check page no. 09, line no. 194 to 195 and page no. 11, line no. 232 to page no. 12, line no. 245. The limitations section is moved to appear before the conclusion as you had suggested, please check page no. 17, line no. 344 to 349.

According to the journal requirement, all the references have been revised carefully. In the first round of revision of the manuscript, irrelevant references were removed and only relevant references were kept and added. In this round of revision, we have carefully checked that no changes and retractions have been made to the references. Currently, the references which have been used all are available and not retracted.

In the first revised version, we removed and added some of our references which were not mistakenly elaborated in previous rebuttal letter and now those are added here,

| Reference | Status | Comment |
| --- | --- | --- |
| Zgheib, Nathalie & Arabi, Asma & Mahfouz, Rami & El-Hajj Fuleihan, Ghada. (2013). OC002—Cyp2r1 Genetic Polymorphisms Are Associated With Lower 25-Hydroxy Vitamin D Levels In Lebanese Subjects. Clinical Therapeutics. 35. e1. 10.1016/j.clinthera.2013.07.003. | Removed | The information for which it was that had been modified and the modified information is available in no.15 article of the current version. |
| Holick MF, Binkley NC, Bischoff-Ferrari HA, Gordon CM, Hanley DA, Heaney RP, et al. Evaluation, treatment, and prevention of vitamin D deficiency: An endocrine society clinical practice guideline. J Clin Endocrinol Metab. 2011;96: 1911–1930. doi:10.1210/jc.2011-0385 | added | It was used to define the refence value of serum 25(OH)D level, (ref no.4 in current version) |
| Binkley N, Ramamurthy R, Krueger D. Low vitamin D status: Definition, prevalence, consequences, and correction. Endocrinol Metab Clin North Am. 2010;39: 287–301. doi:10.1016/j.ecl.2010.02.008 | added | It was used to define the low serum 25(OH)D level, (ref no.5 in current version) |
| Ahmed B, Shiraji KH, Chowdhury MHK, Uddin MG, Islam SN, Hossain S. Socio-economic Status of the Patients with Acute Coronary Syndrome: Data from a District-level General Hospital of Bangladesh. Cardiovasc J. 2017;10: 17–20. doi:10.3329/cardio.v10i1.34357 | added | It was used to determine the socioeconomic status of the study subjects, (ref no.16 in current version) |
| Islam MA, Alam MS, Sharker KK, Nandi SK. Estimation of Solar Radiation on Horizontal and Tilted Surface over Bangladesh. Comput Water, Energy, Environ Eng. 2016;05: 54–69. doi:10.4236/cweee.2016.52006 | added | It was used to determine sun exposure according to geographic location of Bangladesh (ref no.27 in current version) |

Finally, the reference numbers were rearranged according to the article rearrangement during revision.

Responses to Reviewer#3

- You did not discuss the relevance of the significant differences in the biochemical parameters like sgpt, calcium, PT between subjects and controls. What were the reference values for these parameters? Or they were both in the normal ranges for both groups that’s why they were not discussed anymore?

Response: Thank you so much for your concern to make a better version of this article. All the biochemical parameters were within the normal ranges among both the study population and the controls, so they were not discussed anymore. Here, we have added the chart that we followed to ensure the normal range of all the biochemical parameters.

**Reference values of different parameters**

| **Parameters** | **Normal range** |
| --- | --- |
| 1. Serum calcium | 8.50-10.30 mg/dl (Automate  Biochemistry Analyzer) |
| 2. Serum albumin | 3.40 – 5.00 gm/dl (Automated Biochemistry Analyzer) |
| 3. Fasting blood glucose | 3.30 – 6.11 mmol/L (Automated  Biochemistry Analyzer) |
| 4. Serum Creatinine | Male: 0.60 ‑ 1.50  Female: 0.40 ‑ 1.20  Child: 0.20 ‑ 0.70 (Automated Biochemistry Analyzer) |
| 5. Serum SGPT | < 40 U/L (Automated Biochemistry  Analyzer) |
| 6. Serum 25(OH)D | Deficiency:<20ng/ml  Insufficiency: 20-29.9ng/ml  Sufficiency: ≥30ng/ml |

7. Prothrombin time	11-16 second.

- Clarification: were your subjects all healthy with incidental finding of low vitamin D levels? pls state that they were all healthy “meaning no medical problem?”

Response: Thank you for your concern. Yes, all the subjects were healthy and based on your suggestion we have added this clarification in the methodology. Please check page no.5, line no.111.

- I also wanted to know what is the implication in terms of management. Will you recommend vitamin D supplement to those who have low levels? If you will not give supplement, again what is the significance of the study if you will not be able to translate it to clinical practice? Likewise, did you consider checking if the subjects had concomitant disorders associated with low serum vitamin D level? In your introduction you said that low vitamin D is related to several disorders. How can you correlate it with your result- did you ask the medical histories of your subjects and check if these could be related to low vitamin D? (or are they all healthy?)

Response: Thank you for your concern. We began the process by taking the medical history of the study subjects. Their medical history did not reveal any pathological conditions related to low serum 25(OH)D level. In addition to this, we investigated serum calcium level to determine any abnormalities associated with calcium homeostasis. We had a financial constraint for which we were not able to assess serum PTH and other investigations which might be associated with any vitamin D related abnormalities. Since it was a pilot study, the sample size was small. For better results, we need to perform this study on a larger scale. In this pilot study, we have observed that people with low serum 25(OH)D level can perform their physiological functions without any hardship and it is evidenced by the investigation results and also by the history of having no vitamin D related disorders. As all the study subjects were apparently healthy individuals, so it can be hypothesized that due to natural selection, which could be based on the geographical location and sun exposure, variants of rs10741657 (GG, GA) can perform their physiological functions with low serum 25(OH)D level without any need for any kind of vitamin D supplements. But again, to come to a better conclusion, we need further studies with larger sample size and other vitamin D related biochemical analysis. The major implication of this study is to consider the genetic influence in order to redefine the cutoff value of low serum vitamin D level. All these are explained elaborately in the discussion and the conclusion portion of the manuscript, please check page no.16, 17 and 18.

- Will you also recommend to test for this polymorphism routinely among the population? If yes, what is the significance. Will this be harmful for their health?

Response: Since it was a pilot study with a small sample size, we need to further assess to confirm the genetic influence of this polymorphism on vitamin D level on a larger scale. Only then we can recommend if this polymorphism can routinely be done among the population or not. If the precautions are properly taken, it will not be harmful for the health. It has been added in the current version please check page no.17, line no.339 to 342.

- For a better organization, I suggest created subheadings in your methodology. First discuss about the ‘Study Subjects’ the “biochemical analysis” and lastly “molecular analysis”. Do not lump them altogether.

Response: Thank you for your concern, these have been done in the current version. Please check page no. 5 line no. 116, page no. 6 line no. 132 and page no. 7 line no. 141.

Thank you very much for considering our manuscript for revision.

Yours Sincerely,

Kazi Lutfar Rahman

Email: [klrahman@yahoo.com](mailto:klrahman@yahoo.com)
